# Analysis of Total Knee Arthroplasty revision causes

**DOI:** 10.1186/s12891-018-1977-y

**Published:** 2018-02-14

**Authors:** Anne Postler, Cornelia Lützner, Franziska Beyer, Eric Tille, Jörg Lützner

**Affiliations:** University Center of Orthopaedics and Traumatology, University Medicine Carl Gustav Carus Dresden, TU Dresden, Fetscherst. 74, 01307 Dresden, Germany

**Keywords:** Total knee arthroplasty, Failure, Revision, Complication, Re-revision

## Abstract

**Background:**

The number of revision Total Knee Arthroplasty (TKA) is rising in many countries. The aim of this study was the prospective assessment of the underlying causes leading to revision TKA in a tertiary care hospital and the comparison of those reasons with previously published data.

**Methods:**

In this study patients who had revision TKA between 2010 and 2015 were prospectively included. Revision causes were categorized using all available information from patients’ records including preoperative diagnostics, intraoperative findings as well as the results of the periprosthetic tissue analysis. According to previous studies patients were divided into early (up to 2 years) and late revision (more than 2 years). Additional also re-revisions after already performed revision TKA were included.

**Results:**

We assessed 312 patients who underwent 402 revision TKA, 89.6% of them were referred to our center for revision surgery. In 289 patients (71.9%) this was the first revision surgery after primary TKA. Among the first revisions the majority was late revisions (73.7%). One hundred thirteen patients (28.1%) had already had one or more revision surgeries before. Overall, the most frequent reason for revision was infection (36.1%) followed by aseptic loosening (21.9%) and periprosthetic fracture (13.7%).

**Conclusions:**

In a specialized arthroplasty center periprosthetic joint infection (PJI) was the most common reason for revision and re-revision TKA. This is in contrast to population-based registry data and has consequences on costs as well as on success rates in such centers.

## Background

Total knee arthroplasty (TKA) is one of the most frequent surgical procedures and a very effective treatment option for advanced osteoarthritis of the knee, which decreases pain and improves function [1]. Nevertheless, some patients achieve poor results after surgery or the implant fails and a revision surgery is required. The number of revision TKA is rising in many countries, with 22,403 procedures in the United States [2], 15,232 in Australia [3], 5873 in the UK [4] and 17,677 in Germany in 2015 [5].

Previous studies investigated the failure causes after total knee arthroplasties and differed between early (within the first 2 years after primary TKA) and late revision (thereafter). They found polyethylene wear and accordingly aseptic loosening as most common causes for late revisions [6, 7]. Infection and instability were the most common revision causes in the early failure groups [8]. Over the last decade failure mechanisms have changed and polyethylene wear as revision cause decreased. Infection on the contrary was increasing [9, 10]. General information on TKA survival and revision causes in large populations can be obtained from Arthroplasty registries or health care provider data. However, these data are not very specific and provided from many different persons who might have different judgements for categorizing the revision causes [2–4, 11]. Therefore single- or multi-center studies with the possibility to review the patients’ records give a more detailed picture of the revision causes.

The aim of this study was therefore the prospective assessment of causes for revision TKA, and comparison of those reasons with previously published data.

## Methods

After receiving institutional review board approval, all revision surgeries of TKA from January 2010 to December 2015 in our department were prospectively included. According to previous studies we defined revision TKA as replacement of at least one component (femur, tibia or patella), patients with isolated exchange of the polyethylene insert were excluded. Additional and in contrast to other studies we did not only include first revisions, as we intended to present the complete perspective of a tertiary care hospital. The cause of revision was based on analysis of x-rays, Computed tomography (CT) scans, blood tests, joint aspiration, intraoperative findings, culture and histology results. The revision cause was determined by the surgeon in the OR report but was reviewed by the authors using all available data and the below described definitions. Causes were assessed in detail and categorized into infection, aseptic loosening, polyethylene wear, instability, periprosthetic fracture, pain, restricted range of motion/fibrosis, extensor mechanism insufficiency, implant failure and allergy against implant materials. In case of more than one causes for revision the leading cause was reported. The following hierarchy was used: infection, fracture, implant failure, loosening, osteolysis, wear, instability, restricted range of motion, extensor mechanism insufficiency, allergy and pain.

The diagnosis of PJI was based on the Musculoskeletal Infection Society criteria [12]: as two positive periprosthetic cultures with phenotypically identical organisms, or a sinus tract communicating with the joint or having three of the following minor criteria: elevated serum c-reactive protein (CRP), elevated synovial fluid white blood cell count, elevated synovial fluid polymorphonuclear neutrophil percentage, positive histological analysis of periprosthetic tissue or a single positive culture. A two- or multiple-stage revision with temporary placement of an antibiotic-loaded bonecement spacer was always performed and considered as one surgery. Periprosthetic fractures, mechanical implant failure and aseptic loosening were assessed radiographically and if necessary with computed tomography. Polyethylene wear was assessed by macroscopic findings on the insert and microscopic report according to criteria described by Krenn et al. [13]. Instability, pain and extensor mechanism insufficiency were clinical diagnoses by positive history and suitable physical examination. Pain was used as a revision cause only if no other reason could be determined. Fibrosis was defined as a limited range of motion (ROM) in flexion and/or extension, that is not attributable to an osseous or prosthetic block to movement from malaligned, malpositioned or incorrectly sized components, metal hardware, ligament reconstruction, infection, pain, chronic regional pain syndrome (CRPS) or other specific causes, but due to soft-tissue fibrosis that was not present pre-operatively [14]. Allergy against implant materials was considered as revision cause if other reasons could be ruled out (especially PJI), the patients had a positive patch test and the microscopic report described it as likely [13].

Revisions were categorized into two groups: first revision after primary TKA and re-revision after already performed revision surgery. The interval from primary TKA to the first revision surgery was recorded and the patients were categorized into early and late failure groups. An interval between primary TKA and revision procedure of 2 years was considered as the cut-off between early and late failures [7]. In case of more than one revision surgeries (re-revisions) the total number of previous revisions and the time from the last revision was recorded.

Patients demographics, age and gender were documented, as well as Body Mass Index (BMI) and comorbidities (ASA score). Previous surgeries and additional reoperations between implant removal and re-implantation PJI were recorded, too.

Data description is based on means, standard deviations (SD) and ranges for continuous values and absolute and relative frequencies for categorical values. Differences between groups were analyzed using t-test for continuous values and chi-square test for categorical values. A *p*-value of 0.05 results was considered statistically significant. All data analyses were carried out using SPSS (release 22.0 for Windows).

An ethics approval for this study was obtained from the Ethics Committee of the University Medicine Carl Gustav Carus, TU Dresden in 2011 (EK 288082011).

## Results

The study group consisted of 402 cases in 312 patients who had revision TKA between January 2010 and December 2015.The primary TKA was performed in only 42 patients (10.4%) at our department, 360 patients (89.6%) were referred to our center for revision.

There were 402 TKA revisions in 312 patients. Thirty-two patients have been revised bilaterally (64 surgeries) and 25 knees have been revised more than one time during the investigation period. Three hundred thirteen patients had just one surgery. However, we analyzed TKA.

In 289 surgeries (71.9%) this was the first revision surgery in mean 6.2 years (range 0.1 – 24.2 years) after primary TKA. Among the first revisions the majority were late revisions after 2 years (*n* = 207; 73.7%). Three hundred thirteen patients (28.1%) had already had one to 18 (median 1) revision surgeries before. In these patients the time between primary implantation and current re-revision was 3.9 years (range 0.1 – 17.6 years). The overall time to revision 5.5 years (range 0.03 – 24.2 years).

Our patients were in mean 72.3 years old (SD 9.7, range 48.2 – 95.4 years), the majority (64.4%) had severe systemic diseases (ASA grade III and IV) and a mean BMI of 30.6, see Table 1. The 146 patients (36.3%) suffering from infection (72.8 ± 8.8 years; range 48.2 – 92.4 years) were in mean 3 years older than those with aseptic reasons (72.8 vs 69.9 years, *p* = 0.06).

**Table 1:** Comparison of patients characteristics between first and re-revisions

Patients		All revision surgeries (*n* = 402)	First revision surgeries (*n* = 289)	Re-revision surgeries (*n* = 113)	p
Gender	Male	157 (39.1%)	109 (37.7%)	48 (42.5%)	n.s.
	Female	245 (60.9%)	180 (62.3%)	65 (57.5%)	n.s.
Age [years]		72.3 (48.2 – 95.4)	72.2 (48.2 - 95.4)	72.6 (54.4 - 92.5)	n.s.
ASA classification	1	3 (0.7%)	3 (1.0%)	0 (0%)	n.s.
	2	140 (34.8%)	107 (37.0%)	33 (29.2%)	
	3	249 (61.9%)	170 (58.8%)	79 (69,9%)	
	4	10 (2.5%)	9 (3.1%)	1 (0.9%)	
BMI [kg/m^2^]		30.6 (SD 5.7)	30.5 (SD 5.8)	30.9 (5.4)	n.s.

*ASA* classification of the American Society of Anesthesiologists, *BMI* body mass index

**Fig. 1:**
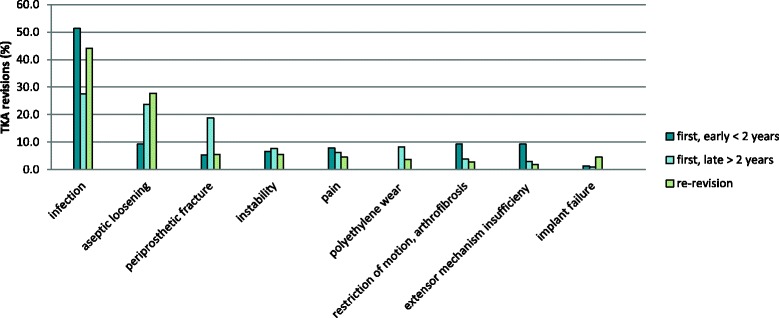
Revision causes according to the type of revision

The most frequent reason for revision was infection (overall 36.1%, first early revisions 51.3%, first late revisions 26.8%, re-revisions 44.2%) followed by aseptic loosening (overall 21.9%, first early revisions 9.2%, first late revisions 23.0%, re-revisions 27.4%) and periprosthetic fracture (overall 13.7%, first early revisions 5.3%, first late revisions 21.1%, re-revisions 5.3%), see Fig. 1 and Table 2 for details. Forty-five patients (11.2%) had just one cause of failure, 356 patients (88.6%) more than one.

**Table 2:** causes and time to revision

Failure mechanism	Total [n (%)] *n* = 402	Time to revision^a^ (yr)	Primary revision [n (%)]				Re-revision	
			Early *n* = 76	Time to revision^a^ (yr)	Late *n* = 207	Time to revision^a^ (yr)	[n (%)]	Time to revision^a^ (yr)
Infection	146 (36.3)	4.2 ± 4.4 (0.06 - 20.1)	39 (51.3)	1.0 ± 0.6 (0.06 - 2.0)	57 (27.5)	7.3 ± 4.8 (2.1 - 20.1)	50 (44.2)	3.3 ± 3.3 (0.1 - 13.0)
Aseptic loosening	87 (21.6)	6.5 ± 5.4 (0.4 - 21.6)	7 (9.2)	1.6 ± 0.2 (1.4 - 2.0)	49 (23.7)	8.6 ± 5.6 (2.1 - 21.6)	31 (27.7)	4.3 ± 3.8 (0.4 - 17.1)
Periprosthetic fracture	55 (13.7)	8.5 ± 5.4 (0.04 - 24.2)	4 (5.3)	0.5 ± 0.6 (0.04 - 1.4)	39 (18.8)	9.5 ± 5.1 (2.6 - 24.2)	6 (5.4)	7.9 ± 4.9 (3.9 - 17.6)
Instability	27 (6.7)	5.4 ± 4.1 (0.08 - 15.2)	5 (6.6)	1.3 ± 0.2 (1.1 - 1.6)	16 (7.7)	6.9 ± 4.1 (2.2 - 15.2)	6 (5.4)	4.9 ± 3.3 (0.1 - 9.2)
Pain	24 (6.0)	3.6 ± 3.0 (1.1 - 11.1)	6 (7.9)	1.4 ± 0.2 (1.1 - 1.6)	13 (6.3)	5.6 ± 3.1 (2.0 - 11.1)	5 (4.5)	1.9 ± 0.4 (1.4 - 2.2)
Polyethylene wear	21 (5.2)	10.5 ± 5.8 (1.6 - 19.5)	0 (0)		17 (8.2)	11.9 ± 5.3 (3.7 - 19.5)	4 (3.6)	4.8 ± 4.2 (1.6 - 10.8)
Restriction of motion, arthrofibrosis	18 (4.5)	2.6 ± 2.3 (0.04 - 9.6)	7 (9.2)	0.9 ± 0.6 (0.04 - 0.9)	8 (3.9)	4.1 ± 2.6 (2.1 - 9.6)	3 (2.7)	2.6 ± 1.9 (0.6 - 4.3)
Extensor mechanism insufficiency	15 (3.7)	2.9 ± 3.6 (0.03 - 11.8)	7 (9.2)	0.7 ± 0.8 (0.03 - 1.8)	6 (2.9)	5.6 ± 4.4 (2.0 - 11.8)	2 (1.8)	2.9 ± 1.9 (1.6 - 4.2)
Mechanically defect	8 (2.0)	5.5 ± 2.8 (1.4 - 9.8)	1 (1.3)	1.4	2 (1.0)	8.5 ± 1.8 (7.2 - 9.8)	5 (4.5)	5.1 ± 2.1 (2.6 - 7.9)
Allergy	1 (0.2)	1.2	0 (0)		0 (0)		1 (0.9)	1.2
Total		5.5 ± 5.0 (0.03 - 24.2)		1.0 ± 0.6 (0.03 - 2.0)		8.1 ± 5.1 (2.0 - 24.2)		3.9 ± 3.5 (0.08 - 17.6)

^a^ values are given as mean ± SD (range)

If the cause for re-revision was PJI (49 patients) the majority (40 patients, 81.6%) had already had one or more revision surgeries due to previous infection.

## Discussion

Total Knee Arthroplasty is a very effective and safe treatment option for advanced osteoarthritis of the knee [15]. Revision rates are generally low, the revision risk at 10 years as reported in the major Arthroplasty registries is about 5%: the Australian Joint Replacement Report stated 5.5% revision rate after 10 years [3], for the UK reported rates are below 5% [4] and slightly more than 5.5% for Sweden [11]. Caused by a growing number of TKA overall, a shift towards younger patients and technical developments of revision implants the number of revisions is growing. While primary TKA is a standard procedure revision TKA is a complex surgery which is often performed in specialized centers. The majority of all revisions in this study (90%) were referred to our center after primary TKA performed elsewhere. Through the concentration of difficult surgeries the causes for revision in specialized centers are likely to be different from population based registries.

In this study the mean time between index surgery and revision TKA of the late revision group was 8.1 years. This is consistent with previous studies, Sharkey et al. reported in 212 with late revisions (more than 2 years) an average time to failure of 7 years (range, 2.2 – 28 years) [7]. Thiele et al. reported a mean time to failure of 7.9 years in patients with a revision of at least 3 years after primary surgery in overall 358 patients [10].

**Fig. 2:**
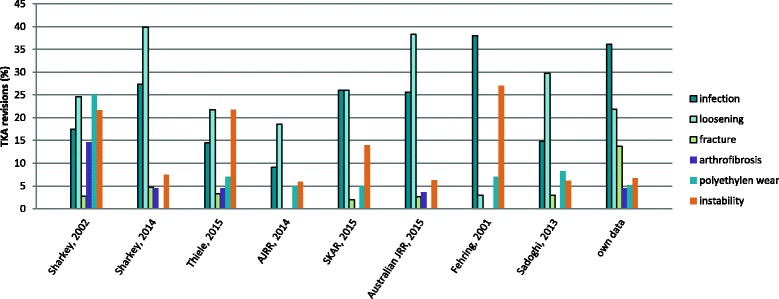
Comparing revision causes between our study and the cited data

The revision causes however, are different to published data, see Fig. 2. In 2002 Sharkey et al. found polyethylene wear (25%) as the most prevalent mechanism for TKA revision [7] and in 2014 aseptic loosening (39.9%) was the most common failure mechanism [9]. Thiele et al. identified aseptic loosening (21.8%), instability (21.8%) and malalignement (20.7%) as the most common indications for revision [10]. In the registries of the US (18.6%), Sweden (about 26%) and Australia (38.3%) aseptic loosening is the most frequent reason for revision, followed by infection (9.1%, about 26%, 25.6%). In our center PJI was the most common reason for revision TKA. The reason for the high frequency of PJI in our patients (146, 36.3%) might be that this is the most difficult to treat and most expensive complication after TKA. Aseptic loosening and other mechanical revision causes might have been considered as “simple” revisions and performed in smaller hospitals whereas PJI were more frequently referred to our department. This has implications on success rates as well as on costs. Success rates for revision TKA for PJI are generally lower than for aseptic revisions [16, 17]. Additionally these revisions have a higher risk of complications with significantly higher length of hospitalization, higher number of readmission and higher rates of mortality [16, 18, 19]. This could be a problem for such centers in pay-for-performance programs which are already in place or intended from different health care providers. Furthermore, revisions for PJI need much more resources and, depending on the health care system, costs are not always completely compensated. Therefore centers performing many revisions for PJI might have disadvantages in public reports on success rates and complications as well as financially. However, PJI are the most challenging complications after TKA and should be treated in specialized centers with experience and sufficient resources [12, 18, 20].

With regard to early surgeries (within 2 years) in the first revisions, the main reason for revision was again infection followed by aseptic loosening, restriction of ROM/arthrofibrosis and extensor mechanism insufficiency just as reported by Sharkey [9] and Thiele [10] for the early revisions. Early loosening of the prosthesis could be related to TKA component fixation methods including cementing technique [9]. Reasons for loosening change with time, which means loosening in the first few years most likely reflects failure to gain fixation. Loosening reported in later years is often due to loss of fixation by secondary bone resorption [3].

For the re-revisions infection (44.2%) and aseptic loosening (27.4%) were the major causes of failure, too. Leta reported about 145 re-revisions after 1016 aseptic revisions in the Norwegian Arthroplasty Register and found deep infection the most frequent cause (28%) because of increased risk after multiple operations, longer operative time, previous scars, larger implants, comorbidities and poorly vascularized tissue [21]. Mortazavi [13] included 499 patients with TKA revisions with a 20.4% re-revision rate and found infection to be the major reason for reoperation or re-revision of the failed TKA (44.1%). Of these periprosthetic joint infections only 32% had no history of infection before, 58% had already had revision due to infection. In our patients 81.6% of patients with re-revision due to PJI had already had revision due to PJI before. These numbers emphasize the difficulties in the treatment of PJI. These patients with re-revisions are more difficult to treat for several reasons. There are often compromised soft-tissues which increases the risk of wound healing problems, because of the restricted circulation. Even adequate exposure might be a problem due to contracture, patella baja and intraarticular scarring. In many of these patients there are already relevant bone defects and consecutively large revision implants sometimes even mega-prostheses are needed. Again, this needs to be taken into consideration when success rates between centers are compared and pay-for-performance programs are being implemented.

We acknowledge some limitations of this study. Most patients were referred to our department and we had therefore not always complete baseline information on the primary TKA. Furthermore we did not always know the precise time to failure. Time to failure is usually less. However, patients with an indication for revision are usually efficiently referred to our center and time between recognized failure and revision is usually less than 3 month. We were not in all cases able to get detailed information about all previous revisions. In some cases, more than one reason lead to revision and we categorized the patients into the leading revision cause. Finally, this is a selection of probably more complicated revision TKA and therefore the frequencies differ from other studies and joint replacement registries. However, this is a more detailed description because we had not only limited information like in registries or from health care provider data. We believe that our data are representative for tertiary care centers.

## Conclusions

Most patients which are revised in a specialized arthroplasty center were referred from other hospitals. PJI was the most common reason for revision and re-revision TKA. This is in contrast to population-based registry data and has consequences on costs as well as on success rates in such centers.

## Abbreviations

ASA score: Physical classification system of the American society of anesthesiologists
BMI: Body mass index
CRP: C-reactive protein
CRPS: Chronic regional pain syndrome
CT: Computed tomography
PJI: Periprosthetic joint infection
ROM: Range of motion
SD: Standard deviations
TKA: Total Knee Replacement
